# Scandium Ion-Promoted Electron-Transfer Disproportionation of 2-Phenyl-4,4,5,5-tetramethylimidazoline-1-oxyl 3-Oxide (PTIO^•^) in Acetonitrile and Its Regeneration Induced by Water

**DOI:** 10.3390/ijms25084417

**Published:** 2024-04-17

**Authors:** Yoshimi Shoji, Yuri Terashima, Kei Ohkubo, Hiromu Ito, Kouichi Maruyama, Shunichi Fukuzumi, Ikuo Nakanishi

**Affiliations:** 1 Quantum RedOx Chemistry Team, Institute for Quantum Life Science (iQLS), Quantum Life and Medical Science Directorate (QLMS), National Institutes for Quantum Science and Technology (QST), Chiba-shi 263-8555, Chiba, Japan; shoji.yoshimi@qst.go.jp (Y.S.); ito.hiromu@qst.go.jp (H.I.); 2 Environmental Radiation Effects Research Group, Department of Radiation Measurement and Dose Assessment, Institute for Radiological Science (NIRS), Quantum Life and Medical Science Directorate (QLMS), National Institutes for Quantum Science and Technology (QST), Chiba-shi 263-8555, Chiba, Japan; terashima.yuri@qst.go.jp (Y.T.); maruyama.kouichi@qst.go.jp (K.M.); 3 Institute for Advanced Co-Creation Studies, Open and Transdisciplinary Research Initiatives, Osaka University, Suita 565-0871, Osaka, Japan; ohkubo@irdd.osaka-u.ac.jp; 4 Department of Chemistry and Nano Science, Ewha Womans University, Seoul 03760, Republic of Korea; fukuzumi@chem.eng.osaka-u.ac.jp; 5 Department of Chemistry, Faculty of Pure and Applied Sciences, University of Tsukuba, Tsukuba 305-8571, Ibaraki, Japan

**Keywords:** radical, electron transfer, disproportionation, scandium ion, Lewis acid, comproportionation, kinetics, reaction mechanism, cyclic voltammetry, electron paramagnetic resonance

## Abstract

2-Phenyl-4,4,5,5-tetramethylimidazoline-1-oxyl 3-oxide (PTIO^•^), a persistent nitronyl nitroxide radical, has been used for the detection and trapping of nitric oxide, as a redox mediator for batteries, for the activity estimation of antioxidants, and so on. However, there is no report on the reactivity of PTIO^•^ in the presence of redox-inactive metal ions. In this study, it is demonstrated that the addition of scandium triflate, Sc(OTf)_3_ (OTf = OSO_2_CF_3_), to an acetonitrile (MeCN) solution of PTIO^•^ resulted in an electron-transfer disproportionation to generate the corresponding cation (PTIO^+^) and anion (PTIO^−^), the latter of which is suggested to be stabilized by Sc^3+^ to form [(PTIO)Sc]^2+^. The decay of the absorption band at 361 nm due to PTIO^•^, monitored using a stopped-flow technique, obeyed second-order kinetics. The second-order rate constant for the disproportionation, thus determined, increased with increasing the Sc(OTf)_3_ concentration to reach a constant value. A drastic change in the cyclic voltammogram recorded for PTIO^•^ in deaerated MeCN containing 0.10 M Bu_4_NClO_4_ was also observed upon addition of Sc(OTf)_3_, suggesting that the large positive shift of the one-electron reduction potential of PTIO^•^ (equivalent to the one-electron oxidation potential of PTIO^−^) in the presence of Sc(OTf)_3_ may result in the disproportionation. When H_2_O was added to the PTIO^•^–Sc(OTf)_3_ system in deaerated MeCN, PTIO^•^ was completely regenerated. It is suggested that the complex formation of Sc^3+^ with H_2_O may weaken the interaction between PTIO^−^ and Sc^3+^, leading to electron-transfer comproportionation to regenerate PTIO^•^. The reversible disproportionation of PTIO^•^ was also confirmed by electron paramagnetic resonance (EPR) spectroscopy.

## 1. Introduction

Redox-inactive metal ions have attracted much attention because they are known to affect the redox behavior of redox active compounds acting as a Lewis acid [1,2,3,4,5,6,7,8,9]. Among such metal ions, scandium ion (Sc^3+^) shows the strongest Lewis acidity because of the small ionic radius with trivalent positive charge, leading to a significant positive shift of the reduction potentials of compounds [10,11,12,13,14,15,16,17,18,19,20,21,22,23], including metal–oxygen complexes [19,20,21] and radical species [22,23]. Of special interest is the fact that Sc^3+^ enables the electron-transfer reactions, which would otherwise never take place in the absence of Sc^3+^, to occur thermodynamically as well as kinetically [10,11,12,13,14,15,20,21]. We have reported that an electron-transfer disproportionation of 2,2-diphenyl-1-picrylhydrazyl radical (DPPH^•^) occurs upon the addition of scandium triflate, Sc(OTf)_3_ (OTf = OSO_2_CF_3_), to an acetonitrile (MeCN) solution of DPPH^•^ to produce one-electron-oxidized and -reduced species of DPPH^•^, DPPH^+^, and DPPH^−^, respectively [23]. The spectral titration showed that four molecules of DPPH^•^ react with Sc^3+^ to produce two molecules of DPPH^+^ and a complex between two molecules of DPPH^−^ and Sc^3+^, [(DPPH)_2_Sc]^+^ [24]. Further, it is also reported that the addition of H_2_O to the DPPH^•^–Sc(OTf)_3_ system in MeCN resulted in the regeneration of DPPH^•^ [24]. Very recently, a similar reversible disproportionation reaction has been reported for 2,2,6,6-tetramethylpiperidyl-1-oxyl (TEMPO) in the presence of Al(OTf)_3_ to develop an aqueous aluminum radical batteries [25]. Dedzo et al. have also reported drastic changes in the redox reactivity of DPPH^•^ in the presence of metal cations, such as Cu^2+^ and Zn^2+^, as well as Brønsted acid, such as HClO_4_ and HNO_3_ [8].

**Scheme 1:**
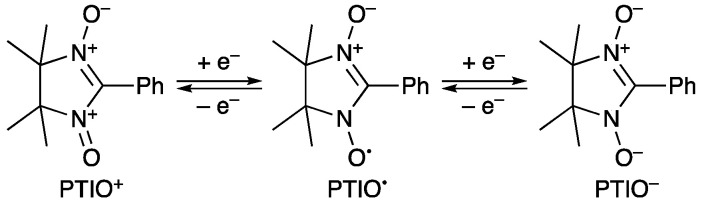
Redox behavior of PTIO^•^.

On the other hand, 2-phenyl-4,4,5,5-tetramethylimidazoline-1-oxyl 3-oxide (PTIO^•^) (Scheme 1), a persistent nitronyl nitroxide radical, has been used for the detection and trapping of nitric oxide (^•^NO) for about 30 years [26,27,28,29,30,31,32,33,34]. Furthermore, PTIO^•^ has also attracted much attention as a redox mediator for batteries [35,36,37]. Recently, it has been reported that PTIO^•^ can be used to estimate the activity of antioxidants as a reactivity model of reactive oxygen species [38,39,40,41,42,43,44,45,46,47,48,49,50,51,52,53,54,55,56,57]. However, there is no report on the reactivity of PTIO^•^ in the presence of redox-inactive metal ions. PTIO^•^ shows a similar reversible one-electron redox behavior (Scheme 1) to DPPH^•^, although the separation between the one-electron oxidation and reduction potentials of PTIO^•^ (1.73 V) [35] is much larger than that of DPPH^•^ (0.52 V) [23]. We report herein that an electron-transfer disproportionation of PTIO^•^ also occurs upon the addition of Sc(OTf)_3_ to a deaerated MeCN solution of PTIO^•^. Also studied was the regeneration of PTIO^•^ upon the addition of H_2_O to the PTIO^•^–Sc(OTf)_3_ system in deaerated MeCN. The drastic change in the redox behavior of compounds due to the strong Lewis acidity of Sc^3+^ observed in this study provides valuable and fundamental information about the fine tuning of the redox reactions by the redox-inactive metal ions.

## 2. Results and Discussion

**Scheme 2:**
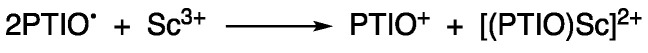
Sc^3+^-promoted electron-transfer disproportionation of PTIO^•^.

**Figure 1:**
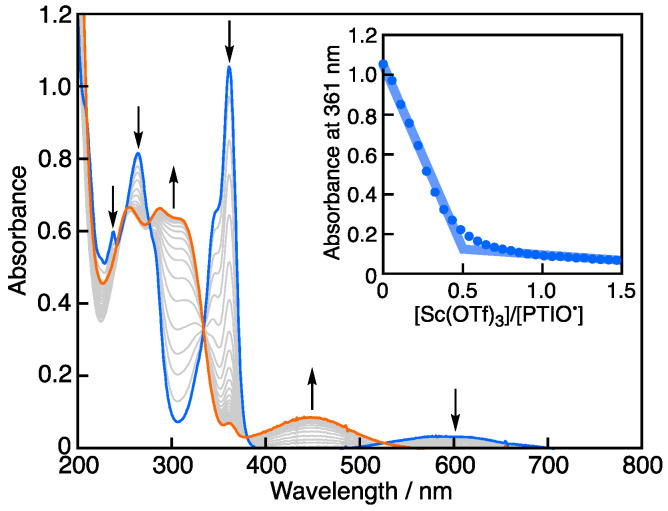
Spectral change observed upon addition of Sc(OTf)_3_ (4.4 × 10^−6^ M each) to PTIO^•^ (7.2 × 10^−5^ M) in deaerated MeCN. Blue and orange lines are the initial and final spectra, respectively. Arrows denote the direction of absorption changes. Inset: plot of the absorbance at 361 nm vs. [Sc(OTf)_3_]/[PTIO^•^].

When Sc(OTf)_3_ was added to a deaerated MeCN solution of PTIO^•^, the absorption bands at 238, 264, and 361 nm and a broad band at around 600 nm due to PTIO^•^ decreased immediately with clear isosbestic points at 277, 334, 381, and 525 nm, as shown in Figure 1 (Video S1 of the Supplementary Materials). The broad absorption band at 450 nm is diagnostic of the one-electron-oxidized PTIO^•^ (PTIO^+^) [33]. Thus, an electron-transfer disproportionation of PTIO^•^ is suggested to take place upon addition of Sc(OTf)_3_ to produce PTIO^+^ and the one-electron-reduced PTIO^•^ (PTIO^−^), as in the case of DPPH^•^ [23]. The spectral titration (inset of Figure 1) shows the Sc(OTf)_3_/PTIO^•^ molar ratio being 1:2. This suggests that one molecule of PTIO^−^ may be stabilized by one Sc^3+^ (Scheme 2). When MeCN was replaced by methanol (MeOH) or ethanol (EtOH) as the solvent, such a spectral change was not observed. This suggests that the stronger solvation of Sc^3+^ in MeOH or EtOH compared to that in MeCN may preclude the disproportionation from occurring.

**Figure 2:**
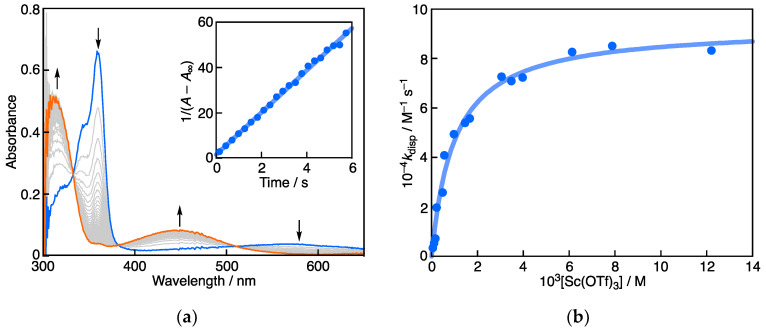
(**a**) Spectral change (interval: 70 ms) observed during the reaction of PTIO^•^ (5.5 × 10^−5^ M) with Sc(OTf)_3_ (7.9 × 10^−3^ M) in deaerated MeCN at 298 K. Blue and orange lines are the initial and final spectra, respectively. Arrows denote the direction of absorption changes. Inset: the second-order plot of the absorbance at 361 nm. (**b**) Plot of *k*_disp_ vs. [Sc(OTf)_3_] for the disproportionation of PTIO^•^ (5.5 × 10^−5^ M) in the presence of Sc(OTf)_3_ in deaerated MeCN at 298 K.

**Scheme 3:**
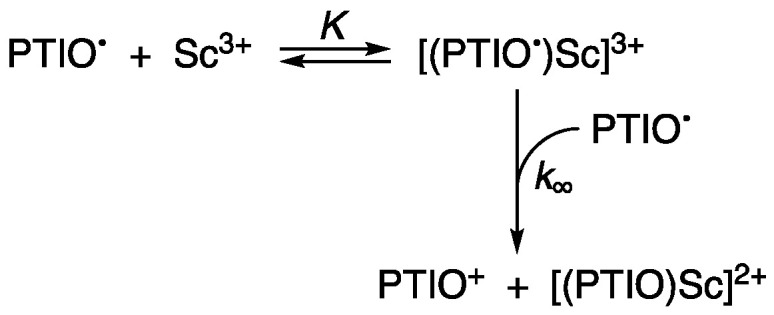
Proposed mechanism of the Sc^3+^-promoted electron-transfer disproportionation of PTIO^•^.

The spectral change after the addition of Sc(OTf)_3_ (7.9 × 10^−3^ M) to a deaerated MeCN solution of PTIO^•^ (5.5 × 10^−5^ M) monitored by a stopped-flow technique is shown in Figure 2a. The time course change in the absorbance at 361 nm obeyed second-order kinetics (inset of Figure 2a). The observed second-order rate constant (*k*_disp_, disp: disproportionation) was determined by a decrease in absorbance at 361 nm due to PTIO^•^. The *k*_disp_ value increases with increasing concentration of Sc(OTf)_3_ ([Sc(OTf)_3_]) to reach a constant value (Figure 2b). The limiting *k*_disp_ value (*k*_∞_) and the binding constant (*K*) between PTIO^•^ and Sc^3+^ were determined from curve fitting based on Scheme 3 and Equation (1) to be 9.3 × 10^4^ M^−1^ s^−1^ and 1.0 × 10^3^ M^−1^, respectively.

According to Equation (1), when [Sc(OTf)_3_] is low (1 >> *K*[Sc(OTf)_3_]), the *k*_disp_ value increases with increasing [Sc(OTf)_3_]. On the other hand, when [Sc(OTf)_3_] is high enough to produce [(PTIO^•^)Sc]^3+^, the reaction between PTIO^•^ and [(PTIO^•^)Sc]^3+^ becomes the rate-determining step and the *k*_disp_ value reaches *k*_∞_.

*k*_disp_ = *k*_∞_*K*[Sc(OTf)_3_]/(1 + *K*[Sc(OTf)_3_]) (1)

**Figure 3:**
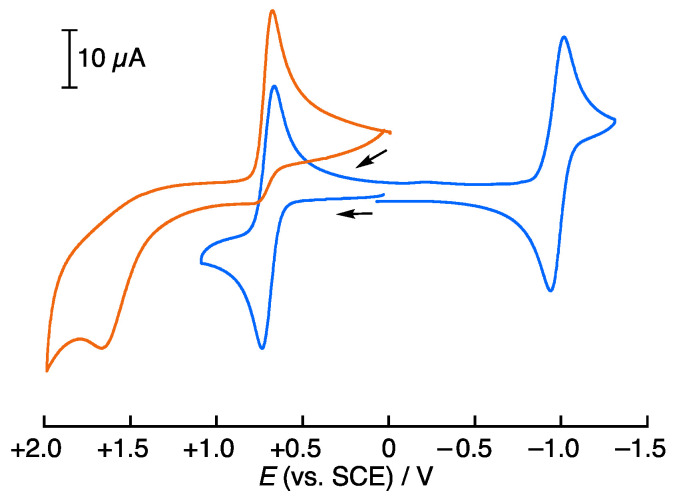
Cyclic voltammograms of PTIO^•^ (1.0 × 10^−3^ M) before (blue line) and after (orange line) the addition of Sc(OTf)_3_ (1.0 × 10^−2^ M) in deaerated MeCN containing 0.10 M Bu_4_NClO_4_ recorded at the scan rate of 100 mV s^−1^ on a glassy carbon working electrode at 298 K. Arrows denote starting points and the direction of scanning.

**Scheme 4:**
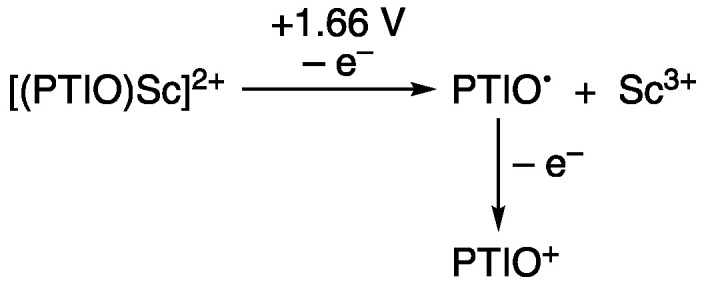
Electrochemical oxidation of [(PTIO)Sc]^2+^ at +1.66 V vs. SCE.

Cyclic voltammetry measurements were carried out to examine the effect of Sc^3+^ on the redox behavior of PTIO^•^ in deaerated MeCN containing 0.10 M Bu_4_NClO_4_. Two well-defined reversible redox waves were observed at −0.98 and +0.70 V vs. the saturated calomel electrode (SCE) for the one-electron reduction and oxidation of PTIO^•^ to produce PTIO^−^ and PTIO^+^, respectively, in the absence of Sc^3+^ (Figure 3). Thus, the separation between the one-electron oxidation and reduction potentials obtained in this study (1.68 V) is slightly smaller than the literature value (1.73 V) [35]. Upon the addition of 10 equiv. of Sc(OTf)_3_, however, a drastic change was observed in the cyclic voltammogram (Figure 3). The reversible wave for the one-electron reduction of PTIO^•^ and the oxidation wave of PTIO^•^ disappeared, while a new oxidation peak appeared at +1.66 V vs. SCE. This new peak was assigned to the oxidation of [(PTIO)Sc]^2+^, which was generated by the disproportionation of PTIO^•^ upon the addition of Sc^3+^, to produce PTIO^•^ and Sc^3+^ (Scheme 4). Then, PTIO^•^ was further oxidized to PTIO^+^ (Scheme 4). Although the reduction peak of PTIO^•^ in the presence of Sc(OTf)_3_ could not be observed due to the disproportionation reaction, such a large (ca. 2.6 V) positive shift of the one-electron reduction potential of PTIO^•^ (equivalent to the oxidation peak for PTIO^−^) upon the addition of Sc(OTf)_3_ enables the disproportionation to occur.

**Figure 4:**
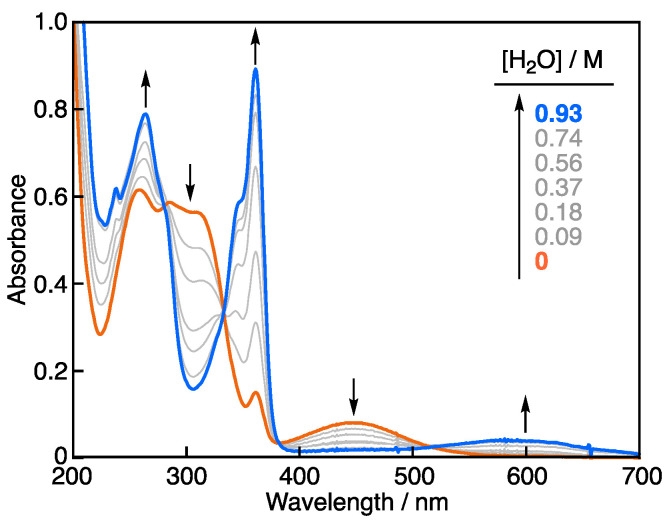
Spectral change upon the addition of H_2_O to a deaerated MeCN solution of PTIO^•^ (7.3 × 10^−5^ M) and Sc(OTf)_3_ (3.7 × 10^−5^ M) at 298 K. Orange and blue lines are the initial and final spectra, respectively. Arrows denote the direction of absorption changes.

The reversibility of the disproportionation of PTIO^•^ by the addition of Sc(OTf)_3_ has also been examined as in the case of DPPH^•^ [24]. The addition of H_2_O to the PTIO^•^–Sc(OTf)_3_ system in deaerated MeCN resulted in the increase in the absorption band at 361 nm due to PTIO^•^ (Figure 4) (Video S2 of the Supplementary Materials). This indicates that an electron-transfer comproportionation occurred to regenerate PTIO^•^. When H_2_O was replaced by EtOH or MeOH, the regeneration of PTIO^•^ was also observed. However, the amount of the recovery was significantly lower compared to the case of H_2_O.

**Figure 5:**
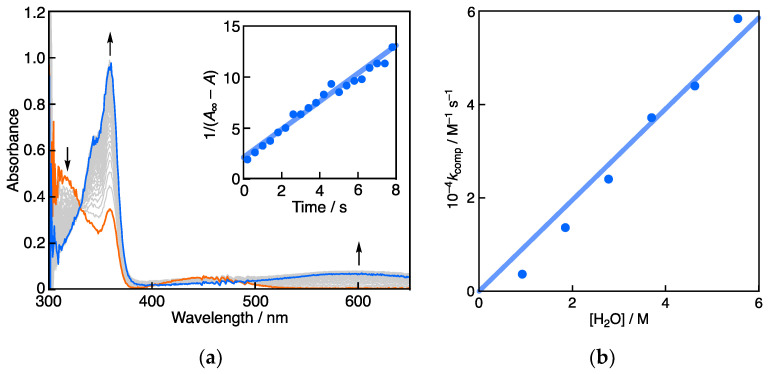
(**a**) Spectral change (interval: 0.1 s) observed after the addition of H_2_O (1.9 M) to a deaerated MeCN solution of PTIO^•^ (6.9 × 10^−5^ M) and Sc(OTf)_3_ (3.5 × 10^−5^ M) at 298 K. Orange and blue lines are the initial and final spectra, respectively. Arrows denote the direction of absorption changes. Inset: the second-order plot of the absorbance at 361 nm. (**b**) Plot of *k*_comp_ vs. [H_2_O].

**Scheme 5:**
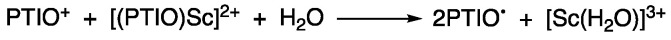
H_2_O-induced regeneration of PTIO^•^.

The spectral change after the addition of H_2_O (1.9 M) to a deaerated MeCN solution containing PTIO^•^ (6.9 × 10^−5^ M) and Sc(OTf)_3_ (3.5 × 10^−5^ M) monitored by a stopped-flow technique is shown in Figure 5a. The time course change in the absorbance at 361 nm obeyed second-order kinetics (inset of Figure 5a), from which the observed second-order rate constant for the comproportionation (*k*_comp_, comp: comproportionation) was determined to be 1.4 × 10^4^ M^−1^ s^−1^. The comproportionation reactions between reduced and oxidized forms of nitroxyl radicals, hydroxyl amines, and oxoammonium cations, respectively, have been extensively studied [58,59,60,61,62,63,64,65,66]. Goldstein et al. determined the *k*_comp_ value between the hydroxylamine and oxoammonium cation derived from TEMPO to be 5.2 × 10 M^−1^ s^−1^ in a phosphate-buffered solution, while the deprotonation of the hydroxylamine resulted in a significant increase in the *k*_comp_ value (3.3 × 10^4^ M^−1^ s^−1^) [59]. This suggests that the electron donor to PTIO^+^ in this study is PTIO^−^, rather than its protonated form (PTIOH). The *k*_comp_ value linearly increased with the increasing concentration of H_2_O, as shown in Figure 5b. Thus, the H_2_O-induced comproportionation reaction is shown in Scheme 5. It is suggested that the complex formation of Sc^3+^ with H_2_O may weaken the interaction between PTIO^−^ and Sc^3+^, leading to the electron-transfer comproportionation regenerating PTIO^•^.

**Figure 6:**
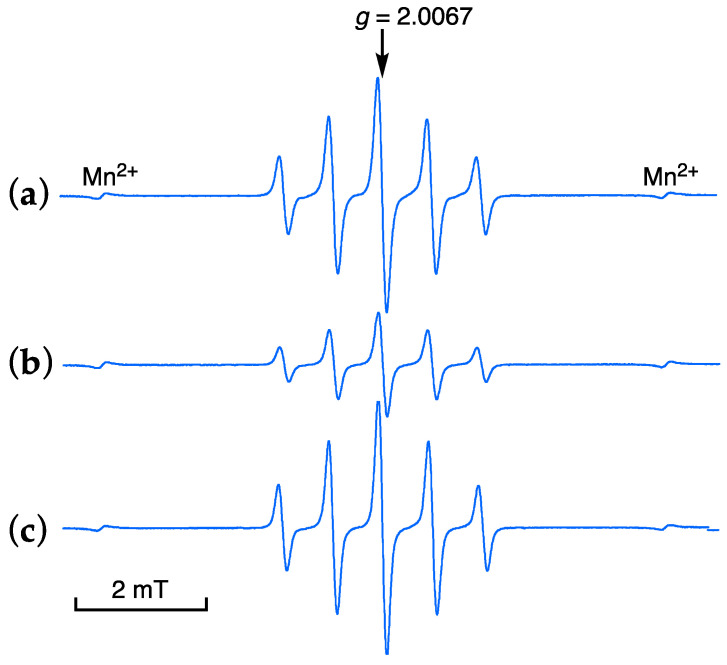
EPR spectra of (**a**) PTIO^•^ (7.0 × 10^−5^ M), (**b**) PTIO^•^ (7.0 × 10^−5^ M) after the addition of Sc(OTf)_3_ (3.5 × 10^−5^ M), and (**c**) PTIO^•^ (7.0 × 10^−5^ M) after the addition of Sc(OTf)_3_ (3.5 × 10^−5^ M) and H_2_O (5.6 M) in deaerated MeCN at room temperature.

The reversible disproportionation of PTIO^•^ was also confirmed by the electron paramagnetic resonance (EPR) spectroscopy. The well-resolved five lines with a *g* value of 2.0067 and a hyperfine coupling constant (*a*_N_) of 0.75 mT were observed in the EPR spectrum of PTIO^•^ in deaerated MeCN (Figure 6a). After 0.5 equiv. of Sc(OTf)_3_ was added to the MeCN solution of PTIO^•^, the signal intensity was significantly decreased, as shown in Figure 6b, although a trace amount of PTIO^•^ was observed. The addition of H_2_O to this PTIO^•^–Sc(OTf)_3_ system in deaerated MeCN resulted in the regeneration of PTIO^•^, which was confirmed by the increase in the EPR signal intensity due to PTIO^•^ (Figure 6c).

## 3. Materials and Methods

### 3.1. Materials

PTIO^•^ was commercially obtained from Tokyo Chemical Industry Co., Ltd., Tokyo, Japan. Sc(OTf)_3_ was purchased from Sigma-Aldrich, St. Louis, MO, USA. MeCN, MeOH, and EtOH (spectral grade) used as solvents were commercially obtained from Nacalai Tesque, Inc., Kyoto, Japan, and used as received. Tetra-*n*-butylammonium perchlorate (Bu_4_NClO_4_), used as a supporting electrolyte for electrochemical measurements, was purchased from Tokyo Chemical Industry Co., Ltd., Tokyo, Japan, recrystallized from EtOH (spectral grade, Nacalai Tesque, Inc., Kyoto, Japan), and dried under vacuum at 313 K. The water used in this study was freshly prepared with a Milli-Q system (Millipore Direct-Q UV3) (Merch Millipore, Burlington, MA, USA).

### 3.2. Spectral and Kinetic Measurements

To avoid the effect of molecular oxygen (O_2_), the reactions were carried out under strictly deaerated conditions, where a continuous flow of argon (Ar) gas was bubbled through each MeCN solution. Typically, a 10 µL of aliquot of Sc(OTf)_3_ (1.3 × 10^−3^ M) in deaerated MeCN was added to a quartz cuvette (10 mm i.d.), which contained PTIO^•^ (7.2 × 10^−3^ M) in deaerated MeCN. UV-vis spectral changes associated with the reaction were monitored using an Agilent 8453 photodiode array spectrophotometer thermostated with a Peltier temperature control at 298 K (Agilent Technologies, Santa Clara, CA, USA). The reaction rates were followed by monitoring the absorbance at 361 nm due to PTIO^•^ after mixing of PTIO^•^ in deaerated MeCN with a deaerated MeCN solution containing Sc(OTf)_3_ at a volumetric ratio of 1:1 using a stopped-flow technique on a UNISOKU RSP-1000-02NM stopped-flow spectrophotometer (UNISOK Co., Ltd., Osaka, Japan), which was thermostated with a Thermo Scientific NESLAB RTE-7 Circulating Bath (Thermo Fisher Scientific, Inc., Waltham, MA, USA) at 298 K. For the regeneration reaction of PTIO^•^, a deaerated MeCN solution containing H_2_O were mixed with a deaerated MeCN solution of PTIO^•^ (1.4 × 10^−4^ M) and Sc(OTf)_3_ (7.0 × 10^−5^ M) at a volumetric ratio of 1:1 using a stopped-flow technique. The observed second-order rate constants (*k*_disp_ and *k*_comp_) were obtained by a least-square curve fit using an Apple MacBook Pro personal computer (Apple Inc., Cupertino, CA, USA) or an HP EliteDesk 800 G4 SFF (HP Inc., Palo Alto, CA, USA). The plots of 1/(*A* − *A*_∞_) vs. time (*A* and *A*_∞_ are the absorbance at the reaction time and the final absorbance, respectively) were linear until three or more half-lives, with a correlation coefficient ρ > 0.999. The *k*_disp_ and *k*_comp_ values were calculated by Slope(*A*_0_ − *A*_∞_)/[PTIO^•^]_0_, where Slope is the slope of the linear plot of 1/(*A* − *A*_∞_) vs. time, and *A*_0_ and [PTIO^•^]_0_ are the initial absorbance at 361 nm and initial concentration of PTIO^•^, respectively. In each case, it was confirmed that the *k*_disp_ and *k*_comp_ values derived from at least three independent measurements agreed within experimental error of ±5%.

### 3.3. Electrochemical Measurements

The cyclic voltammetry measurements were performed on an ALS-630A electrochemical analyzer (BAS Co., Ltd., Tokyo, Japan) in deaerated MeCN containing 0.10 M Bu_4_NClO_4_ as a supporting electrolyte. The continuous flow of Ar gas was bubbled through each MeCN solution to avoid the effect of O_2_. The glassy carbon working electrode (3 mm diameter) (BAS Co., Ltd., Tokyo, Japan) was polished with polishing alumina suspension (BAS Co., Ltd., Tokyo, Japan) and an alumina polishing pad (BAS Co., Ltd., Tokyo, Japan) and rinsed with methanol (FUJIFILM Wako Pure Chemical Corporation, Osaka, Japan) prior to each measurement. The counter electrode was a platinum wire (BAS Co., Ltd., Tokyo, Japan). The concentration of PTIO^•^ and Sc(OTf)_3_ were 1.0 × 10^−3^ and 1.0 × 10^−2^ M, respectively. The measured potentials were recorded with respect to an Ag/AgNO_3_ (0.01 M) reference electrode (BAS Co., Ltd., Tokyo, Japan) with a sweep rate of 100 mV s^−1^ at 298 K. The potentials were converted to those vs. the saturated calomel electrode (SCE) by adding 0.29 V [67].

### 3.4. EPR Measurements

The EPR spectra of PTIO^•^ (7.0 × 10^−5^ M) in the presence or absence of Sc(OTf)_3_ (3.5 × 10^−5^ M) and/or H_2_O (5.6 M) in deaerated MeCN were taken using a disposable RDC-60-S flat cell (inner size, 60 mm × 6 mm × 0.3 mm) (Flashpoint Ltd., Tokyo, Japan) on a JEOL X-band spectrometer (JES-RE1X) (JEOL Ltd., Tokyo, Japan) at room temperature under the following conditions: microwave frequency, 9.40 GHz; microwave power, 8 mW; center field, 333 mT; sweep width, 15 mT; sweep rate, 3 mT min^−1^; modulation frequency, 100 kHz; modulation amplitude, 0.2 mT; and time constant, 0.1 s. EPR data acquisition was controlled by the WIN-RAD ESR Sata Analyzer System (Radical Research, Inc., Tokyo, Japan). The *g* values were calibrated with an Mn^2+^ marker. The experimental EPR spectra were analyzed and simulated using the WinSim 2002 software [68].

## 4. Conclusions

The Sc^3+^ with a strong Lewis acidity induced the electron-transfer disproportionation of PTIO^•^ in deaerated MeCN. The electrochemical measurements suggested that the significantly large positive shift of the one-electron reduction potential of PTIO^•^ in the presence of Sc^3+^ enables the disproportionation to occur. The addition of H_2_O to the PTIO^•^–Sc(OTf)_3_ system in deaerated MeCN resulted in the regeneration of PTIO^•^ because the complex formation of Sc^3+^ with H_2_O weakened the interaction between PTIO^−^ and Sc^3+^. The drastic change in the redox reactivity of PTIO^•^ in the presence of Sc^3+^ as a strong Lewis acid provides not only valuable and fundamental information about the effects of the reaction environments on the reactivity of radical species but an excellent opportunity to develop radical-based redox flow batteries.

## Data Availability

Data are contained within the article.

## Supplementary Materials

The following supporting information can be downloaded at: https://www.mdpi.com/article/10.3390/ijms25084417/s1.

## Abbreviations

Al(OTf)_3_	Aluminum triflate (OTf = OSO_2_CF_3_)
Bu_4_NClO_4_	Tetra-*n*-butylammonium perchlorate
DPPH^•^	2,2-Diphenyl-1-picrylhydrazyl
DPPH^−^	One-electron-reduced species of DPPH^•^
DPPH^+^	One-electron-oxidized species of DPPH^•^
EPR	Electron paramagnetic resonance
EtOH	Ethanol
MeCN	Acetonitrile
MeOH	Methanol
^•^NO	Nitric oxide
PTIO^•^	2-Phenyl-4,4,5,5-tetramethylimidazoline-1-oxyl 3-oxide
PTIO^−^	One-electron-reduced species of PTIO^•^
PTIO^+^	One-electron-oxidized species of PTIO^•^
PTIOH	Protonated form of PTIO^−^
SCE	Saturated calomel electrode
Sc(OTf)_3_	Scandium triflate (OTf = OSO_2_CF_3_)
TEMPO	2,2,6,6-Tetramethylpiperidyl-1-oxyl
